# In reply: acute intraventricular obstruction as a complementary trigger of takotsubo cardiomyopathy in endurance athletes

**DOI:** 10.21542/gcsp.2026.37

**Published:** 2026-08-31

**Authors:** Husam Katib, Ngoc Thai Kieu, Sabeeh Islam

**Affiliations:** University at Buffalo, Jacobs School of Medicine and Biomedical Sciences, Department of Internal Medicine, Buffalo, NY, USA

Dear Editor,

We thank Yalta et al. for their thoughtful and timely Letter concerning our review of takotsubo cardiomyopathy (TTC) in endurance athletes^1,2^. Their emphasis on acute intraventricular obstruction as a mechanical trigger of TTC—distinct from, yet potentially complementary to, catecholamine-mediated myocardial stunning and microvascular dysfunction—raises an important and underrecognized dimension of this condition in athletes.

As our review was a structured synthesis of published case reports and series rather than a primary cohort with investigator-collected imaging, our conclusions were necessarily bound by the granularity of the echocardiographic and cardiac MRI data reported in the source literature. Across the 42 cases analyzed, septal wall thickness, resting or provoked intraventricular gradients, and dedicated provocation testing (e.g., dobutamine stress echocardiography, Valsalva maneuvers) were inconsistently documented, and in most cases not reported at all. This precluded any systematic assessment of how frequently mechanically-triggered TTC, as opposed to the classic catecholamine-microvascular pathway, may account for TTC in this population. We agree with Yalta et al. that this represents a meaningful gap in the existing literature rather than evidence against a mechanical contribution.

We also agree that the phenotypic overlap between physiological athlete’s heart and mild pathological hypertrophy (including borderline hypertrophic cardiomyopathy, hypertensive heart disease, and ventricular septal bulge) is a clinically important diagnostic pitfall, particularly among older or masters-level endurance athletes^3^. Hemodynamic stressors common to marathon and ultra-endurance participation, including dehydration, post-exercise vagotonia, and surges in myocardial contractility, could plausibly unmask a latent gradient in athletes with an underlying, previously unrecognized structural predisposition. We view this mechanism as additive to, rather than competing with, the catecholamine and microvascular pathways emphasized in our review, and it is plausible that both operate together in at least a subset of cases.

The point raised regarding recurrence risk is particularly relevant to this population. Unlike the broader TTC population, endurance athletes with a mechanically-triggered episode may be more likely to re-encounter the same hemodynamic conditions upon returning to training and competition, which has direct implications for return-to-play counseling and surveillance^4^. We agree this warrants dedicated attention in future studies.

We welcome the perspective offered by Yalta et al. as a valuable complement to our review and would encourage future prospective, athlete-focused TTC studies to incorporate structured septal geometry assessment and provocation testing where clinically indicated. Such data would help clarify the true prevalence of mechanically-triggered TTC in this population and better inform diagnosis, risk stratification, and return-to-activity decisions.
